# Metals in the pathogenesis of type 2 diabetes

**DOI:** 10.1186/2251-6581-13-16

**Published:** 2014-01-08

**Authors:** Abdul Rehman Khan, Fazli Rabbi Awan

**Affiliations:** 1 Diabetes and Cardio-Metabolic Disorder (D&C-MD) Laboratory, Health Biotechnology Division, National Institute for Biotechnology and Genetic Engineering (NIBGE), Jhang Road, Faisalabad, P.O. Box.577, Pakistan; 2 Department of Chemistry, University of Azad Jammu and Kashmir, Muzaffarabad, 13100, Pakistan

**Keywords:** Diabetes, Essential metals, Toxic metals, Insulin, Zinc

## Abstract

Minerals are one of the components of food, though they are not synthesized in the body but they are essential for optimal health. Several essential metals are required for the proper functioning of many enzymes, transcriptional factors and proteins important in various biochemical pathways. For example Zn, Mg and Mn are cofactors of hundreds of enzymes, and Zn is involved in the synthesis and secretion of insulin from the pancreatic beta-cells. Similarly, Cr enhances the insulin receptor activity on target tissues, especially in muscle cells. Insulin is the key hormone required to maintain the blood glucose level in normal range. In case of insulin deficiency or resistance, blood glucose concentration exceeds the upper limit of the normal range of 126 mg/dl. Persistent increase of blood serum glucose level leads to overt chronic hyperglycemia, which is a major clinical symptom of diabetes mellitus. Poor glycemic control and diabetes alters the levels of essential trace elements such as Zn, Mg, Mn, Cr, Fe etc. by increasing urinary excretion and their concomitant decrease in the blood. Hence, the main purpose of this review is to discuss the important roles of essential trace elements in normal homeostasis and physiological functioning. Moreover, perturbation of essential trace elements is also discussed in perspective of type 2 diabetes pathobiology.

## Introduction

### Metals and type 2 diabetes (T2D)

Metals are naturally occurring inorganic elements which are present in very small amounts in the living tissues but are important for the vital processes of life [1]. Some metals (e.g. magnesium) are known as macro-metals and are found in high amount in the body tissues, therefore they are also called macro-nutrients [2]. At least 100 mg of each macro-nutrient is required in the daily diet [3]. In contrast, some metals e.g. copper (Cu), zinc (Zn), iron (Fe) and manganese (Mn), chromium (Cr) etc. are needed in the body in very small amounts, less than 100 parts per million (ppm), hence, these are called trace elements or micro-nutrients [4]. Metals are involved in a range of physiological processes such as prosthetic groups of many proteins, water balance, cofactors of many enzymes etc. [5]. Several metals function as part of proteins/enzymes as metalloproteinase/metalloenzymes [6]. Such proteins without metal containing prosthetic groups are unable to perform their physiological functions [7]. The regulation of various metallic contents in the body is pre-requisite for their proper functioning [8]. Metals enable the muscles to contract or relax, and also transmit impulses through the nerves. Most metals are available in the soluble salt forms, which regulate the composition of biofluids. The proper metabolic functioning of the trace elements depends on their normal levels in various body tissues [9]. Due to the diversified metabolic characteristics and functions; various metals such as Mg, Zn, Cr, Fe, Mn and Cu are considered as essential for normal human health [1].

Several studies have reported that the imbalance of some essential metals might adversely affect pancreatic islet and cause development of diabetes [10]. It is also manifested that some reactive oxygen species (ROS) are produced during diabetes due to imbalance of essential metals. This oxidative stress might decrease the insulin gene promoter activity and mRNA expression in pancreatic islet cells due to hyperglycemic condition [11-13].

On contrary to essential metals, some toxic metals have also been identified which accumulate in various biological samples of T2D patients. Uncontrolled pollution and industrialization might be a potential source to expose human population against toxic metals such as lead (Pb), nickel (Ni), cadmium (Cd) and arsenic (As). Some of the toxic metals are implicated to disrupt the glucose uptake and alter the related molecular mechanism in glucose regulation [14,15].

### Essential metals and their physiological roles

#### Iron (Fe)

Iron (Fe) is an essential transition metal required for the synthesis of two important functional proteins such as hemoglobin and myoglobin, which are involved in the transport of molecular oxygen during respiration [16]. It is also required in the elastin production along with Zn and ascorbic acid and collagen synthesis [17]. In blood stream small fraction of serum Fe is transported by a glycoprotein, called transferrin into the cells [18]. In the body tissues, ferritin stores free Fe, which is increased in newly diagnosed diabetic subjects [19,20]. Jiang *et al.* manifested higher level of ferritin in diabetics as compared to the non-diabetic subjects. Recently, a report showed a positive correlation between serum ferritin and Fe deposition in tissues, which linearly increased with diabetes duration [21]. The serum ferritin elevation is regarded as an index of Fe overload, which successively leads to a condition called hemochromatosis [22]. Several studies showed association between hemochromatosis and type 2 diabetes (T2D) [23-25]. The elevated Fe level oxidizes various biomolecules such as nucleic acids, proteins and lipids, which may contribute to T2D development by decreasing insulin secretion from pancreatic beta cells with concomitant increase of insulin resistance [13,26-29]. Previous studies [30,31] manifested a strong relationship between serum ferritin level and insulin resistance at preclinical stage prior to the development of full blown diabetes mellitus. Regarding this, studies also suggested that in addition to the glucose elevation, serum ferritin level might become a surrogate marker of diabetes to predict disease onset [32,33].

#### Magnesium (Mg)

Mg is the most abundant macro-nutrient which is essential for the maintenance of proper health. It is required for the activity of more than 300 enzymes, which serve several important physiological functions in the human body [34]. Mg containing enzymes are involved in the glucose homeostasis, nerve transmission, DNA and RNA production [35].

In prospective cohort studies an association was investigated between Mg consumption through diet and the risk of type 2 diabetes. Furthermore, it was demonstrated that Mg deficiency might lead to a decrease in insulin mediated glucose uptake [34,36]. On the other hand, Mg supplementation prevented insulin resistance and also reduced the development of diabetes in animal models [37]. Some studies reported low level of Mg in the blood serum and an increased urinary excretion of Mg in the diabetics relative to their healthy control subjects [36].

#### Manganese (Mn)

Mn acts as a cofactor in several enzymes including those involved in bone marrow production, and metabolism of carbohydrates, proteins and fats [38]. It is essential for the proper utilization of choline, thiamine, biotin, vitamin C and vitamin E. Mn as a cofactor of enzymes is also involved in mitochondrial glycoproteins synthesis [15].

Impaired activity of these enzymes, due to Mn deficiency leads to abnormal cartilage production [39]. Mn is also a cofactor of pyruvate carboxylase, which plays a role in the conversion of various non-carbohydrate compounds into glucose via gluconeogenesis for their subsequent use. In short, Mn is also required for normal insulin synthesis, its secretion, and an alteration in its metabolism has been implicated in diabetes development [1]. Very recently, in an elegant study by Forte and colleagues reported Mn deficiency in type 2 diabetic patients with respect to their control subjects [40].

#### Copper (Cu)

Cu is another essential mineral, which is needed for several biological functions. It is required for the catalytic activity of superoxide dismutase (SOD) that participates in the protection of cells from superoxide radicals [41]. Cu imbalance is implicated in cholesterol elevation by disrupting normal high density lipoproteins (HDL) and low density lipoproteins (LDL) balance [42]. Cu also activates cytochrome oxidase which is involved in the electron transport chain of the mitochondria [43]. In case of copper deficiency, cytochrome oxidase reduces its activity which might lead to the distortion of mitochondria in metabolically active tissues such as pancreatic acinar cells, hepatocytes etc. [44,45].

Published data show that Cu deficiency is one of the reasons for the development of cardiovascular diseases [46]. Other reports suggest that Cu is also beneficial to prevent arthritis associated inflammation and epilepsy [47]. More recently, it has been reported that disturbances in copper levels in various biofluids and tissues are associated with abnormalities implicated in metabolic pathways of diabetes and its complications [1,48]. Copper as well as zinc metals play roles in order to protect oxidative damage of body tissues [49,50].

#### Zinc (Zn)

Zinc (Zn) is an essential trace element which is required for normal cell processing e.g. cell division and apoptosis. Zn participates in multiple biochemical pathways such as in transcription, translation and cell divisions [51]. More than 300 enzymes need Zn for their catalytic activities. On the other hand, removal of Zn from catalytic site leads to the loss of enzymatic activity [52].

About 70% of the Zn is bound to albumin and any pathological alteration of albumin affects the serum Zn levels [53]. Zn malabsorption results in various types of disorders including the dermal, gastrointestinal, neurological and immunological abnormalities [54].

Recently, published studies revealed that type 2 diabetic patients have suboptimal Zn status in blood due to its increased urinary depletion [1,40]. As a result, hypozincemia and hyperzincuria are developed in diabetics [1,54]. Zn plays a key role in the storage and secretion of insulin, which subsequently increases the uptake of glucose [1,55]. The decreased plasma level of Zn adversely affects the ability of islet cells to produce and secrete insulin [55,56].

It is well established that Zn transporter (ZnT8) is a key protein for the regulation of insulin secretion from the pancreatic β-cells. Recently a mutation in ZnT8 transporter has been associated with T2D [57]. Briefly all these evidences show the importance of Zn in the maintenance and integration of insulin hexamer and its role in the metabolic regulation [55].

#### Chromium (Cr)

The biological activity of Cr depends on its valence state and chemical complexes it forms [9,58,59]. Trivalent form of Cr has high biological activity which is required for the optimal glucose uptake by cells [59,60]. Cr regulates insulin and blood glucose levels by stimulating insulin signaling pathway and metabolism by up-regulating glucose transporter (GLUT4) translocation in muscle cells [61].

Cr deficiency results in the elevation of blood glucose levels and if it is persisted for a prolonged period, it may lead to the development of diabetes [62]. Some reports show that Cr supplements decrease the blood sugar level in diabetes [63]. Prolonged hyperglycemia increases Cr urinary excretion [1,40].

Briefly, cumulative evidences indicate the essential nature of these metals for the maintenance of human normal physiology. While their imbalance predisposes to glucose intolerance which subsequently converts to diabetes related complications [1].

### Toxic metals and health

Toxic metals e.g. lead (Pb), nickel (Ni), cadmium (Cd) and arsenic (As) deposit in tissues and are non-degradable. Hence, these metals remain in the tissues for a long period, and it is often difficult to eliminate metal-based problem. Body tissues can tolerate a certain level of metals, and beyond such threshold limits tissues get damaged due to metal toxicity. Some of the toxic metals including Ni, As etc. are manifested as carcinogens [11,64,65].

The mechanism of metal induced carcinogenesis is elusive, which might be due to complex nature of interactions of metals in biological systems [11]. Furthermore, essential metals also have carcinogenic effects if present in excess amounts than they are required. For example, Cr^3+^ is essential while Cr^4+^ behaves as carcinogenic agent [64]. Similarly, hemochromatosis increases the risk of hepatocarcinoma [66]. Preponderance of these toxic metals in the environment is potentially alarming and harmful for human health [67]. They are common in the nature and present in air, water and soil, which increases the probability of human exposure [15].

Toxic metals react with various proteins in the body that may modify their functions and kinetics. Moreover, when diet is low in essential metals, the body absorbs and makes use of more toxic metals. In the current environmental conditions several human populations are exposed to high levels of toxic metals including Pb, Cd, As and Ni [65]. An abundance of a toxic metal competes with essential metal for enzymes activity and various body physiological functions [68]. For example, Zn is required for the activity of many enzymes. In case of Zn deficiency and increased exposure to toxic metals such as lead (Pb), body will use Pb instead of Zn [69].

Some toxic metals including Pb, Cd, As and Ni are elevated in biological samples of diabetic patients, which adversely affect health status of an individual by disrupting organ physiology and functions. Free Pb in blood plasma is rapidly transferred to soft body tissues [70].

#### Lead (Pb)

Some toxic metals including Pb were reported in higher concentration in the biological samples (i.e. blood plasma and urine) of diabetics than the non-diabetics [71]. Pb is hazardous to most of the human body organs, and interferes with metabolism and cellular functions [72]. Previous reports showed a linear relationship between blood Pb level and renal dysfunction in age-related diseases. This may be due to the frequent exposure to environmental Pb [73].

Studies demonstrated that exposure to Pb badly affects the antioxidant pathways [65]. Existing evidences indicate that metal induced toxicity may cause derangement of antioxidant mechanisms in living tissues; as a consequence highly reactive oxygen species (ROS) are generated. This antioxidant imbalance might lead to the degradation of proteins, nucleic acids and lipid peroxidation. An oxidative attack of cellular components by ROS is implicated in the pathogenesis of several human diseases including diabetes [14,74].

#### Cadmium (Cd)

Cd is a heavy metal which is widely detected in environment such as air, water and soil. Increased Cd level in water is absorbed by plants, animals and humans [70]. Frequent exposure to Cd contributes to its excess accumulation in kidney, which results in renal damage and nephropathy [14]. Furthermore, Cd high level reduces calcium absorption, which becomes an impending cause of bone and kidney losses, called Itai-Itai disease. It has also been reported that Cd might down-regulate glucose transporter-4 (GLUT4) translocation by insulin and enhanced the induction of pancreatic beta cells’ disruption in diabetes [75].

#### Arsenic (As)

Arsenic is a naturally occurring deadly semi-metal, which is mainly used in copper alloys and lead batteries. As is also used in the insecticides, herbicides and pesticides production. Ground water contamination with arsenic is becoming an alarming threat for human lives thoughout the world [11,71]. Frequent exposure to arsenic linked to various diseases including certain types of cancers and diabetes. Previous studies indicate that As implicates in the disruption of glucose metabolism due to an alteration in cell signaling tranduction factors such as tumor necrosis factor-α (TNF-α), mitogen-activated protein kinase (MAPK) etc., subsequently GLUT4 translocation to membrane might be arrested. Some studies manifest the connection of arsenic in beta-cell dysfunction [14,75].

#### Nickel (Ni)

Ni is ferromagnetic element which is mostly used in Ni-Cd batteries. Most of the human populations are exposed to Ni through different means such as drinking polluted water, air and eating nickel contaminated food [76]. It has been observed that kidney is the major organ for Ni accumulation, thus contributes in renal dysfunction [77]. Most recently, Forte et al. found that type 2 diabetic patients have 0.89 ng/ml of Ni in the blood relative to 0.77 ng/ml in the control subjects [40].

Briefly, investigators have analyzed various biofluids such as blood serum/plasma, hair, urine etc. in order to know the altered metabolism of metals in various diseases including diabetes. Among these biological samples, urine was unique because it is readily available and non-invasively sampled. In some previously published studies, researchers have shown urinary levels of metals which correspond to their blood serum status of this metal [1,14]. Aforementioned studies showed the existence of link between the levels of toxic metals and essential trace metals [78].

## Conclusion

From published literature on derangement of metals in diabetes, it could be concluded that normal levels of essential metals are disturbed in T2D patients. The status of essential trace elements in various body tissues is influenced by renal excretion. Alteration in the level of one metal may influence normal levels of other metals. It can also be suggested that toxic metals such as Pb, Ni and Cd may have a role to induce renal tubular dysfunction in diabetic subjects. Subsequently dysfunctional kidneys may become a potential source for the loss of several essential trace elements through urine voiding rather than their retention in blood plasma /serum in order to retain the homeostasis of blood and other tissues. The high urinary excretion of Zn results in its reduced levels in blood plasma/serum which is the sign of disruptions in Zn based antioxidant mechanism. Therefore, in future large population based prospective studies on urinary analysis of various essential and toxic metals, may help to have scientific as well as practical implications in understanding and controlling diabetes or its complications like diabetic nephropathy.

## Competing interests

Authors state that there is no competing interest regarding the publication of this article.

## Authors’ contributions

ARK wrote the initial drafts and FRA revised the manuscript. Both authors read and approved the final manuscript.

## Authors’ information

ARK has background in Chemistry, who did his PhD thesis research in FRA’s lab. ARK has recently submitted his PhD thesis on diabetes research. FRA holds a PhD degree in Biochemistry and working as a Senior Scientist at the Diabetes and Cardio-Metabolic Disorder (D&C-MD) Laboratory, Health Biotechnology Division, National Institute for Biotechnology and Genetic Engineering (NIBGE), Faisalabad, Pakistan. FRA is also affiliated with QAU (Quaid-i-Azam University) and PIEAS (Pakistan Institute of Engineering and Applied Sciences), Islamabad, Pakistan as Adjunct Faculty.
